# The Mediator Role of Organizational Justice in the Relationship Between School Principals’ Agile Leadership Characteristics and Teachers’ Job Satisfaction

**DOI:** 10.3389/fpsyg.2022.895540

**Published:** 2022-07-14

**Authors:** Mustafa Özgenel, Şebnem Yazıcı, Adem Asmaz

**Affiliations:** ^1^Department of Educational Sciences, Faculty of Education, Istanbul Sabahattin Zaim University, Istanbul, Turkey; ^2^Department of Educational Sciences, Faculty of Education, Fatih Sultan Mehmet Vakif University, Istanbul, Turkey; ^3^Educational Administration, Istanbul Sabahattin Zaim University, Istanbul, Turkey

**Keywords:** leadership, agile leadership, job satisfaction, justice, organizational justice

## Abstract

Teachers with high job satisfaction become more effective and productive by showing high performance and positively affecting student and school outcomes. This study investigates the relationship between school principals’ agile leadership characteristics, organizational justice, and job satisfaction, and sheds light on the role of organizational justice in the relationship between agile leadership and job satisfaction. To achieve this aim, a theoretical model has been put forward and this model has been tested with the data collected from 409 teachers working in public schools in Istanbul. The results show that school principals with high agile leadership characteristics and organizational justice are associated with teachers’ job satisfaction. The analyses also indicate that organizational justice strongly contributes to the relationship between agile leadership and job satisfaction and plays an important role in the relationship between the two determinants.

## Introduction

Today, leaders try to deal with elements such as Volatility, Uncertainty, Complexity and Ambiguity (VUCA) (Joiner and Josephs, 2007; De Meuse et al., 2008; Horney et al., 2010). This situation forced organizations to manage complex and dynamic environments and differentiated their understanding of Leadership (Litz, 2011; Alsaeedi and Male, 2013). In order for leaders to be successful in VUCA environments, it is necessary to demonstrate a range of effective behaviors such as competitiveness, readiness for change and effective use of resources. There is a common view that agile leaders, who can use these behaviors effectively and easily manage uncertain environments, will also transform the organization into a more agile structure (Lediju, 2016; Hollis, 2017; Fielitz and Hug, 2019; Gren and Lindman, 2020). Many researchers study agile leadership and school effectiveness (Çalışkan Yılmaz, 2021; Yalçın and Özgenel, 2021), organizational commitment (Lediju, 2016; Kostrad, 2019; Özdemir, 2020) and employee engagement (Fitaloka et al., 2020), organizational learning and innovation (Muafi and Uyun, 2019), gender (Saro, 2017; Akkaya and Üstgörül, 2020; Cestou, 2020), performance (Lediju, 2016; Yalçın and Özgenel, 2021). Focusing on the relationship between organizational agility (Young, 2013; Joiner, 2019), flexibility and adaptability (Hollis, 2017), they tried to reveal the effects of agile leadership on the organization and employees. Since the research on agile leadership is new and limited, more research is needed on this subject with different variables.

Today, educational organizations, like other organizations, are to effectively manage environments that are difficult to control. While educational organizations discuss how to handle VUCA (Akinoso, 2015; Reeves and Reeves, 2015; Stewart et al., 2016), they also focus on the effects of the agile school leader, who can lead these environments, inside and outside the organization (Taylor, 2017; Özdemir, 2020; Özgenel and Yazici, 2020; Çalışkan Yılmaz, 2021). Although these studies try to determine the effects of the agile leader on the employee and the organization, it is seen that there are important gaps in the subject. One of them is the job satisfaction of the employees, which can shape the success of the organization. In many studies conducted on teachers, it has been revealed that different leadership practices and behaviors have positive reflections on job satisfaction (Pool, 1997; Nguni et al., 2006; Hariri et al., 2016; Sun and Xia, 2018; Torlak and Kuzey, 2019; Torres, 2019; Maheshwari, 2021). However, job satisfaction, which is an important factor in ensuring the teacher effectiveness of the agile school leader, has been left as a subject that needs research. In addition, the agile leader’s integrating all stakeholders with the goals of the organization, encouraging cooperation, providing opportunities for new ideas, motivating flexible management approach (Joiner and Josephs, 2007; Joiner, 2009), exhibiting an impartial, fair management style can also strengthen teachers’ perceptions of organizational justice. However, organizational justice is considered important as a factor affecting teachers’ job satisfaction, since it includes evaluating teachers’ feelings about the school environment and the work they do at school, and meta-analysis research results in the literature prove the effect of organizational justice on job satisfaction (Cohen-Charash and Spector, 2001; Korkmaz, 2021). Since the current research findings and theoretical knowledge are insufficient to explain the relationship between agile leadership, organizational justice and job satisfaction, which is the subject of this study, it is aimed to determine whether organizational justice perceptions will mediate the relationship between school principals’ agile leadership characteristics and teachers’ job satisfaction. Determining which factors are effective at school level in terms of improving teachers’ job satisfaction and revealing the relationships between agile leadership, organizational justice and job satisfaction may enable managerial inferences to be made. Discussing the organizational effects of the agile leader by including them in the research, as well as the individual effects of the agile leader, determining how organizational justice mediates, will provide a better understanding of the subject and will improve our understanding of the relationship between these variables.

## Theoretical Background and Hypotheses Development

### The Effect of Agile Leadership on Organizational Justice and Job Satisfaction

The concept of agile leadership is defined as the ability to offer fast and effective solutions in the face of ambiguous and complex situations, adapting their skills to different situations, and displaying flexible behaviors (Joiner and Josephs, 2007; Joiner, 2009). Continuous learning, gaining experience, and willingness to develop, which is suggested as a leadership skill, are among the features frequently used by the agile leader (Lombardo and Eichinger, 2000; Mumford et al., 2000). The ability to develop different solution strategies with experiential learning and to apply them quickly to new situations to improve their changing business skills gives the agile leader the power to manage teams (Joiner and Josephs, 2007; De Meuse et al., 2010). Agile leaders’ effective communication skills, transparent management approach, involving employees in decision-making processes without considering their personal interests, gaining the respect and support of their followers ensure that they are perceived as a fair leader by their followers (Joiner and Josephs, 2007). In addition, agile leaders keeping communication channels open with employees and providing fair rewards and promotion opportunities with the feedback they obtain will create a sense of trust in employees. In this way, it will enable the employees to perceive the organization more justly and the leaders to exhibit a more agile management approach. The sense of organizational justice is considered important as it affects the attitudes and behaviors of employees (Hubbell and Chory-Assad, 2005; Cheng, 2014). Organizational justice is expressed as employees’ perception of whether their behavior toward them is fair or not (Moorman, 1991). Leaders’ management styles affect organizational justice, and this situation is also reflected in educational environments (Uğurlu and Üstüner, 2011). For this reason, it is thought that the management style of agile school leaders will positively affect the perception of organizational justice.

Another important factor for the organization to achieve sustainable success is job satisfaction. Because job satisfaction is the positive emotional response to the extent to which an individual meets the things, he considers important for his job (Locke, 1969; Luthans, 2011) and these reactions direct their performance (Petty et al., 1984). Existing research reveals that the leadership styles of school principals are an important factor in teachers’ positive perception of their work environment and their job satisfaction (Bogler, 2001; Dreer, 2021). While agile school principals increase the organizational commitment of teachers (Özdemir, 2020), they support their professional development and have a positive effect on their performance (Yalçın and Özgenel, 2021). Self-efficacy beliefs of school principals that they can overcome difficult situations enable them to be more persistent and make an effort (Wood and Bandura, 1989). Accordingly, teachers’ job satisfaction increases (Federici and Skaalvik, 2012) and they feel safe (Nielsen et al., 2011). Since agile leaders have high self-efficacy, they can increase teachers’ job satisfaction by taking decisive steps in the face of uncertainty (London and Smither, 1999) and managing uncertainty (Joiner and Josephs, 2007; Horney et al., 2010; Setili, 2015). Given the theoretical and empirical evidence between the variables, we hypothesize the following:

H_1_: *Agile leadership has a significant and positive direct effect on job satisfaction and organizational justice.*

### The Effect of Organizational Justice on Job Satisfaction

Job satisfaction lies under the attitudes of the employees toward the organizational environment (Burke, 2004) and their feelings (Spector, 1997). Locke (1976) defines job satisfaction as a pleasurable or positive emotional state resulting from the appraisal of one’s job or job experiences’ (p. 1304). Organizational researchers have long been working to identify the antecedents and consequences of greatest job satisfaction (Hoppock, 1937; Vroom, 1962; Lawler and Porter, 1967; Wanous and Lawler, 1972; Weaver, 1980; Scarpello and Campbell, 1983; Conway et al., 1987; Arvey et al., 1989; Furnham and Drakeley, 1993; Clark, 1996; Wright and Cropanzano, 2000; Judge et al., 2002, 2012; Crossman and Harris, 2006; Lee et al., 2012; Mondal and Saha, 2017). The main purpose of these studies is to try to eliminate, prevent or improve the factors that negatively affect job satisfaction by determining its antecedents and results. Accordingly, it is aimed to increase the performance of the employees in a specific sense, and to increase the efficiency and effectiveness of the organization in general. Indeed, studies support this idea. For example, it has been revealed that employees with high job satisfaction are productive, have a higher probability of staying in the organization (McNeese-Smith, 1997), has higher performance (Greene, 1972; Iaffaldano and Muchinsky, 1985; Judge et al., 2001, 2002; Harter et al., 2002; Hunter, 2006) and life satisfaction (Tait et al., 1989; Judge and Watanabe, 1993), positively affects organizational outcomes (Faragher et al., 2013), reduce turnover (Steel and Rentsch, 1995), and leave of employment (Griffeth et al., 2000; Judge et al., 2002). However, it has been reported that employees with low job satisfaction are not productive/effective, experience burnout (Faragher et al., 2013), and prevent them from being innovative (Raziq and Maulabakhsh, 2015).

Job satisfaction can be traced back to Hawthorne studies, which sought to understand how employees behave in organizational settings. Determining which organizational characteristics are important in the context of increasing/developing employees’ job satisfaction provides managerial implications (Churchill et al., 1974). Ultimately, it improves organizational effectiveness. It is tried to answer how wage increases, promotions, job status, and similar distributions within the organization, in other words, organizational justice practices affect the attitudes and behaviors of employees (Greenberg, 1987a,b, 1990, 1993). In this context, research on job satisfaction and organizational justice includes evaluating/determining employees’ feelings of satisfaction or dissatisfaction with their work environment and job, and this due diligence affects employee behaviors and organizational outputs (Cohen-Charash and Spector, 2001). Greenberg (1987b) explained organizational justice as the individual’s perception of justice regarding wage increases, promotions, job status, and similar practices in the organization (p. 55). In general, employees’ perceptions of organizational justice are examined in three dimensions: distributive justice, procedural justice, and interactional justice (Greenberg, 1987a,b; Colquitt et al., 2001). Distributive justice refers to the distribution of the gains that employees get such as wages, compensation, and rewards (Daileyl and Kirk, 1992; Clay-Warner et al., 2005; Bahri-Ammari and Bilgihan, 2017), procedural justice is the method used to distribute these gains (Daileyl and Kirk, 1992; Işikay, 2020) and interactional justice is related to whether interpersonal behavior is fair or not while the procedures are applied (Greenberg, 1993; Fischer, 2012). In other words, organizational justice focuses on how employees perceive and react to justice practices in the organization. In the literature, capital research findings are showing that organizational justice perceptions of employees affect their job satisfaction positively and significantly in non-profit service sectors such as education, health, and security (Fatimah et al., 2011; Crow et al., 2012; Nojani et al., 2012; Iqbal, 2013; Lotfi and Pour, 2013; Altahayneh et al., 2014; Chegini et al., 2019; Jameel et al., 2020; Korkmaz, 2021) and for-profit commercial sectors such as energy, insurance, technology, furniture, finance, automotive, and tourism (Daileyl and Kirk, 1992; Martin and Bennett, 1996; Blau and Andersson, 2005; Al-Zu’bi, 2010; Ahmadzadeh Mashinchi et al., 2012; Yelboğa, 2012; López-Cabarcos et al., 2015; Hao et al., 2016; Negahban et al., 2017; Ulutas, 2018; Sung, 2021). To this extent, the job satisfaction of employees decreases or increases depending on whether the organizational justice perception is positive or negative. Given the theoretical and empirical evidence between the variables, we hypothesize the following:

H_2_: *Organizational justice has a significant and positive direct effect on job satisfaction.*

### Indirect Effect of Agile Leadership on Job Satisfaction Through Organizational Justice

Agile leaders can regulate emotions that prevent them from solving problems in times of crisis (Joiner and Josephs, 2007; Yukl and Mahsud, 2010; McCauley et al., 2013). The emotional agility of the leaders, their orientation to feedback to understand the needs and emotional reactions of the employees correctly, enable them to make the right decisions and eliminate the uncertainty (Pescosolido, 2002) and to easily respond to this uncertainty (Joiner and Josephs, 2007). This open flow of information between the leader and the employees also corrects the injustices within the organization, leading to the further development of the perception of justice (Sherf et al., 2021). Organizational justice is an important element in the functioning of schools, as in every organization. While school administrators’ supportive leadership behaviors toward teachers and their fair perception of the organization cause them to trust their administrators (Yilmaz and Altinkurt, 2012), they enable them to be more committed to their profession (Alazmi and Alenezi, 2020). Teachers who perceive the organization as fairer are likely to experience high job satisfaction (Zainalipour et al., 2010; Nojani et al., 2012; Elma, 2013; Ghran et al., 2020). In addition, the mediation effect of organizational justice on the relationship between emotional intelligence and job satisfaction was determined by the research result (Ouyang et al., 2015). As a result, this study argues that school principals’ agile leadership characteristics will be effective in gaining job satisfaction and organizational justice perceptions, which are of great importance for teachers to provide sustainable quality education at school. Given the theoretical and empirical evidence between the variables, we hypothesize the following:

H_3:_ *Agile leadership has a significant and positive indirect effect on job satisfaction through increased organizational justice.*

## Materials and Methods

### Research Model

**FIGURE 1:**
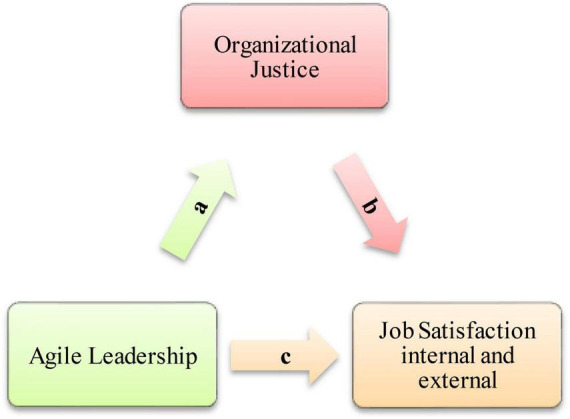
The conceptual framework for the study.

In this research, which aims to determine the mediating role of organizational justice in the relationship between school principals’ agile leadership characteristics and teachers’ job satisfaction, a mediation model was established theoretically (Figure 1) and the mediation model was tested with the collected data. The purpose of the mediation model is to determine the function of a third variable that affects the direction or strength of the independent variable’s effect on the dependent variable (Baron and Kenny, 1986).

According to Figure 1, school principals’ agile leadership characteristics affect teachers’ perceptions of both internal and external job satisfaction and organizational justice. Accordingly, while teachers’ organizational justice perceptions directly affect their job satisfaction, teachers’ organizational perceptions mediate the relationship between school principals’ agile leadership characteristics and teachers’ job satisfaction. According to Hayes (2015), determining the causal effect of a variable (X) on another variable (Y) gives us limited information about the relationship between variables. However, confirming/testing that the relationship between these two variables may affect other variables indicates a deeper understanding than just determining that X affects Y, and such studies contribute a lot to science. According to Baron and Kenny (1986), for a variable to be accepted as a mediating variable, the effect of the independent variable (agile leadership) on the dependent variable (job satisfaction) is added to the model by adding the mediating variable (organizational justice) to the dependent variable (job satisfaction) should decrease to zero. The decrease in the path coefficient between the independent variable (agile leadership) and the dependent variable (job satisfaction) to zero is evidence of a “dominant mediating variable.” However, if the path coefficient between the independent variable (agile leadership) and the dependent variable (job satisfaction) is not zero, this finding indicates that there may be “multiple mediating variables” between the independent variable and the dependent variable.

### Participants

Participants consist of 409 teachers working in public and private schools in the Sancaktepe district of Istanbul in the 2020–2021 academic year. 409 teachers who were involved in the research participated voluntarily, 72.1% of the teachers were female and 27.9% were male. 87.5% of the teachers have bachelor’s degrees and 12.5% have postgraduate degrees. 21% of the teachers work at primary school, 45.5% at secondary school, and 20.3% at high school. Of these 409 teachers, 29.6% have 5 or less seniority, 34.2% have 6–10 years of seniority, 16.1% have 11–15 years of seniority, 9.5% have seniority of 16–20 years, and 10.5% of them have 21 years or more seniority.

### Data Collection Tools

#### Marmara Agile Leadership Scale

The scale, consisting of 34 Likert-type items, is developed by Özgenel and Yazici (2020). The scale consists of three sub-dimensions: situational awareness, human relations, and self-awareness. The 5-point Likert scale was arranged as “Never-0, Rarely-1, Sometimes-2, Often-3, Always-4.” The possible scores for the scale are between 0 and 136. While high scores indicate high agile leadership characteristics; low scores indicate a low level of agile leadership characteristics. The reliability coefficient of the scale was calculated as 0.959 (Özgenel and Yazici, 2020).

#### Minnesota Job Satisfaction Scale

The scale, which consists of 20 Likert-type items and two sub-dimensions, internal satisfaction, and extrinsic satisfaction, is developed by Weiss et al. (1967). The scale was graded as a 5-point Likert type (1-Not at all satisfied, 2-Not satisfied, 3-Not sure, 4-Satisfied, 5-Very Satisfied). Although internal and external satisfaction scores are obtained for the scale, the highest score to be obtained from the scale is 100 and the lowest score is 20. The scale was adapted to Turkish by Baycan (1985), and in this study, the internal consistency coefficient of the Minnesota Job Satisfaction Scale was found to be 0.77.

#### Organizational Justice Scale

The scale consisting of one dimension and 10 items is developed by Hoy and Tarter (2004). The scale is rated as a five-point Likert type (1-Never satisfied, 2-Rarely satisfied, 3-Sometimes/sometimes satisfied, 4-Mostly satisfied, 5-Always satisfied). The adaptation of the scale to Turkish was carried out by Tastan and Yilmaz (2008) and the internal consistency coefficient of the scale was calculated as 0.92.

### Analysis of the Data

The data obtained within the scope of the research were analyzed with SPSS and AMOS package program. First, Skewness and Kurtosis values were examined to determine whether the data showed normality distribution, and reliability coefficients were calculated (Table 1).

**TABLE 1:** The skewness, kurtosis, and reliability values of the scales.

	*N*	*M*	SD	Skewness	Kurtosis	Cronbach’s alpha
Agile leadership	409	2.93	0.67	−0.958	0.434	0.980
Organizational justice	409	4.05	0.62	−0.835	0.418	0.905
Internal satisfaction	409	4.10	0.53	−0.321	0.637	0.876
External satisfaction	409	3.62	0.59	0.249	0.131	0.787

In Table 1, it is seen that the Skewness and Kurtosis values of the scales are in the range of −1 +1. These values revealed that the scores obtained from the scales had a normal distribution. In addition, it is seen that the reliability values of the scales were 0.787 and above, and it is decided that the scales were reliable. Correlation analysis is used to determine the relationship between variables in the analysis of the data, and mediation model analysis is used to test the conceptual model. The mediation analysis proposed by Baron and Kenny (1986) was conducted in three stages.

## Findings

Correlation analysis is performed to determine the relationship between the variables and the result is presented in Table 2.

**TABLE 2:** The relationship between agile leadership, job satisfaction, and organizational justice.

Variables		1	2	3	4	*M*	SD
1. Agile leadership	*r*	–				2.931	0.674
2. Organizational justice	*r*	0.777**	–			4.058	0.631
3. Internal job satisfaction	*r*	0.397**	0.455**	–		4.107	0.535
4. External job satisfaction	*r*	523**	0.575**	0.678**	–	3.622	0.596

N: 409; **p < 0.01.

When Table 2 is examined, it is revealed that there is a positive and high level (*r* = −0.777; *p* < 0.01) relationship between school principals’ agile leadership characteristics perceived by teachers and teachers’ organizational justice perceptions. Meanwhile, a positive and moderate relationship (*r* = 0.397; *p* < 0.01) between agile leadership characteristics and internal job satisfaction is detected. However, it is seen that there is a positive and moderate (*r* = 0.523; *p* < 0.01) significant relationship between agile leadership characteristics and external job satisfaction. After determining the relationships between the variables, the mediator of the independent variable and the dependent variable; The effect of the mediating variable on the dependent variable is calculated.

**FIGURE 2:**
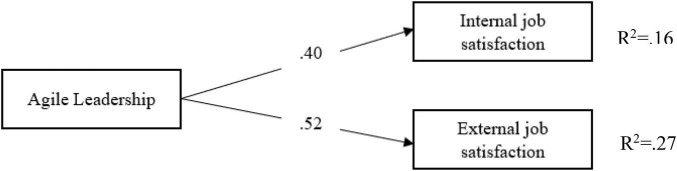
The relationship between agile leadership and job satisfaction.

According to Figure 2, the path coefficients between school principals’ agile leadership characteristics and teachers’ internal job satisfaction (β = 0.40; *p* < 0.05) and external job satisfaction [β = 0.52 (*p* < 0.05)] are significant. School principals’ agile leadership characteristics significantly predict teachers’ internal (*R*^2^ = 0.16; *p* < 0.05) and external job satisfactions (*R*^2^ = 0.52; *p* < 0.05). In other words, school principals’ agile leadership characteristics explain 16% of the total variance in teachers’ internal job satisfaction and 27% of the total variance in external job satisfaction.

**FIGURE 3:**
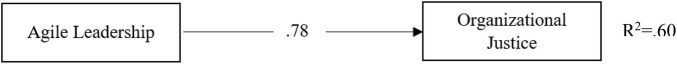
The relationship between agile leadership and organizational justice.

According to Figure 3, it is seen that the path coefficients between school principals’ agile leadership characteristics and teachers’ organizational justice perceptions (β = 0.78; *p* < 0.05) are significant. School principals’ agile leadership characteristics significantly predict teachers’ perceptions of organizational justice (*R*^2^ = 0.60; *p* < 0.05). In other words, school principals’ agile leadership characteristics explain 60% of the total variance in teachers’ perceptions of organizational justice.

**FIGURE 4:**
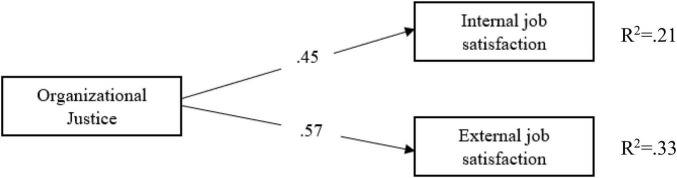
The relationship between organizational justice and job satisfaction.

**FIGURE 5:**
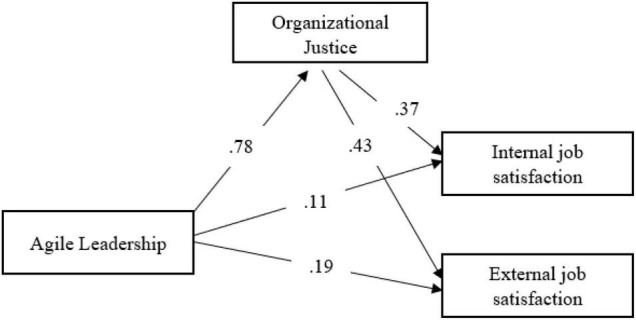
Theoretical model.

According to Figure 4, the path coefficients between teachers’ perceptions of organizational justice and internal (β = 0.45; *p* < 0.05) and external job satisfaction (β = 0.57; *p* < 0.05) are significant. Teachers’ perceptions of organizational justice significantly predicted their internal (*R*^2^ = 0.21; *p* < 0.05) and external job satisfaction (*R*^2^ = 0.33; *p* < 0.05). Teachers’ perceptions of organizational justice explain 21% of the total variance in their internal job satisfaction and 33% of the total variance in their external job satisfaction. After determining that there is a significant relationship between the independent, dependent, and mediator variables, the proposed theoretical model, together with the three variables, was tested by mediation analysis with the collected data (Figure 5).

The proposed theoretical model was analyzed and the path coefficients between the variables were examined. It is seen that the path coefficients between agile leadership and internal and external job satisfaction have decreased significantly. However, to evaluate the model, it is first examined whether the path coefficients between the variables were significant (Table 3).

**TABLE 3:** Regression values between variables.

			Estimate	S.E.	C.R.	*p*
Organizational justice	<–	Agile leadership	0.715	0.029	24,910	***
External job satisfaction	<–	Agile leadership	0.170	0.056	3,021	0.003
Internal job satisfaction	<–	Organizational justice	0.318	0.060	5,297	***
External job satisfaction	<–	Organizational justice	0.408	0.061	6,688	***
Internal job satisfaction	<–	Agile leadership	**0.087**	**0.055**	**1,568**	**0.117**

Bold indicates p-values that are not significant. *p < 0.05, **p < 0.01, ***p < 0.001.

**FIGURE 6:**
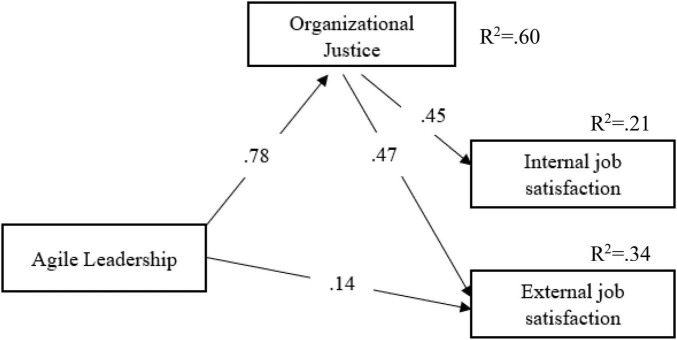
Measurement model.

When Table 3 is examined, agile leadership and organizational justice (β = 0.78; *p* < 0.05) and external job satisfaction (β = 0.19; *p* < 0.05); while a significant relationship was found between organizational justice and internal (β = 0.37; *p* < 0.05) and external (β = 0.43; *p* < 0.05) job satisfaction (*p* < 0.05); it was seen that the path coefficient between agile leadership and internal job satisfaction (β = 0.11; *p* > 0.05) was not significant and the path between agile leadership and internal job satisfaction was excluded from the model and the analysis was re-executed (Figure 6).

When Figure 6 is examined, it is seen that the path coefficient between agile leadership and external job satisfaction (β = 0.14; *p* < 0.05) decreased significantly after the path coefficient between agile leadership and internal job satisfaction was removed. In addition, agile leadership and organizational justice (β = 0.78; *p* < 0.05); it was determined that there is a significant relationship between organizational justice and internal job satisfaction (β = 0.45; *p* < 0.05) and external job satisfaction (β = 0.47; *p* < 0.05) (*p* < 0.05). In other words, while the agile leadership characteristics of school principals alone explain 16% of the total variance in teachers’ internal job satisfaction; Agile leadership and organizational justice together explain 21% of the total variance in teachers’ internal job satisfaction. Again, while the agile leadership characteristics of school principals alone explain 27% of the total variance in teachers’ external job satisfaction; Agile leadership and organizational justice together explain 33% of the total variance in teachers’ external job satisfaction. From these findings, the agile leadership characteristics of school principals, together with organizational justice, increase the positive effect on teachers’ internal and external job satisfaction. To compare the proposed theoretical model with the measurement model, it is checked whether the path (regression) coefficients between the repeat variables are significant (Table 4).

**TABLE 4:** Regression values between variables.

			Estimate	S.E.	C.R.	*p*
Organizational justice	<–	Agile leadership	0.715	0.029	24,910	***
External job satisfaction	<–	Agile leadership	0.120	0.046	2,588	0.010
Internal job satisfaction	<–	Organizational justice	0.392	0.038	10,314	***
External job satisfaction	<–	Organizational justice	0.451	0.055	8,224	***

*p < 0.05, **p < 0.01, ***p < 0.001.

When Table 4 is examined, a significant relationship is discovered between agile leadership and organizational justice, external job satisfaction, organizational justice, and internal and external job satisfaction (*p* < 0.05). To evaluate the model, standardized total, direct, and indirect effect values (Table 5) and fit indices (Table 6) between the variables are examined.

**TABLE 5:** Standardized total, direct and indirect effect values between variables.

Standardized total effects	Agile leadership	Organizational justice
Organizational justice	0.777	0.000
External job satisfaction	0.502	0.471
Internal job satisfaction	0.353	0.455
**Standardized direct effects**		
Organizational justice	0.777	0.000
External job satisfaction	0.136	0.471
Internal job satisfaction	0.000	0.455
**Standardized indirect effects**		
Organizational justice	0.000	0.000
External job satisfaction	0.366	0.000
Internal job satisfaction	0.353	0.000

*T, Total impact; D, vertical/direct impact; I, indirect effect.*

**TABLE 6:** Fit indexes for proposed models.

	χ^2^	*df*	*p*	χ*^2^/df*	RMR	SRMR	GFI	AGFI	NFI	TLI	CFI	RMSEA
Model	2.452	1	0.117	2.452	0.006	0.0153	0.997	0.970	0.997	0.989	998	0.060
Reached values				Perfect	Perfect	Perfect	Perfect	Perfect	Perfect	Perfect	Perfect	Acceptable

*χ^2^, Chi-square; df, degree of freedom; p < 0.01; RMR, Root mean square residuals; SRMR, Standardized root mean square residual; GFI, Goodness-of-fit index; AGFI, Adjusted goodness-of-fit index; NFI, Normed Fit Index; TLI, Tucker–Lewis Index; CFI, Comparative Fit Index; RMSEA, Root mean square error of approximation.*

When Table 5 is scrutinized, standardized total effect values are determined to be significant between school principals’ agile leadership characteristics and organizational justice (β = 0.77; *p* < 0.05), external (β = 0.50; *p* < 0.05), internal (β = 0.35; *p* < 0.05) job satisfaction with organizational justice and external (β = 0.47; *p* < 0.05) and internal (β = 0.45; *p* < 0.05) job satisfaction. Similarly, it is also explored that the standardized direct effect values between school principals’ agile leadership characteristics and organizational justice (β = 0.77; *p* < 0.05) and external job satisfaction (β = 0.13; *p* < 0.05) with organizational justice and external (β = 0.47; *p* < 0.05) and internal (β = 0.45; *p* < 0.05) job satisfaction values. It is also seen that there is a standardized indirect effect between the agile leadership characteristics of school principals and teachers’ external job satisfaction (β = 0.36; *p* < 0.05) and internal job satisfaction (β = 0.35; *p* < 0.05). While organizational justice is the full mediator in the relationship between agile leadership and internal job satisfaction, organizational justice played a “partial mediator” role in the relationship between agile leadership and external job satisfaction. Goodness-of-fit indices were examined to determine the validity of the model (Table 6).

When the goodness of fit indices of the measurement model are examined in Table 6, it is seen that it is a valid model according to the reference intervals recommended in the literature to evaluate structural equation/mediation models (Maydeu-Olivares and Garcia-Forero, 2010; Schumacker and Lomax, 2010; Kline, 2011).

## Discussion

Factors such as Volatility, Uncertainty, Complexity and Ambiguity have changed the understanding of leadership in the business world and an agile leadership approach that will effectively manage these elements has been presented as an answer (Lombardo and Eichinger, 2000; Joiner and Josephs, 2007; Joiner, 2009, 2019; De Meuse et al., 2010). The effects of agile leadership on the organization and employees, especially in the field of education, remain at the theoretical level. Therefore, in this study, we tried to prove the direct and indirect effects of school principals’ agile leadership characteristics on teachers’ job satisfaction and perceptions of organizational justice and to make an empirical contribution to the development of the concept. In the research, the answer to the question of whether organizational justice has a gap role in the relationship between the agile leadership characteristics of school principals and the job satisfaction of teachers was sought. A positive and moderately positive relationship was found between school principals’ agile leadership characteristics perceived by teachers and their internal and external job satisfaction and organizational justice perceptions. In addition, a moderate and positive significant relationship is determined between teachers’ perceptions of organizational justice and their internal and external job satisfaction. In the literature, it has been revealed that job satisfaction is related to different leadership styles (Chiok Foong Loke, 2001; Rad and Yarmohammadian, 2006; Rumph, 2012). Concordantly, agile leaders establish strong communication with employees, exhibit a collaborative approach, receive feedback from employees, and perceive employees’ different ideas as a development tool (Scott et al., 2003; Joiner and Josephs, 2007; Taylor, 2017). It can be said that they try to increase their job satisfaction. In addition to these features, agile leaders develop a sense of belief and confidence in their employees to cope with unfamiliar situations (Cashman, 2011). Leaving a safe space for the employees to express their ideas freely and express their thoughts can develop the idea that leaders can be just. The fair behaviors of their leaders perceived by the employees cause them to think that they are fair in their organization (DeConinck, 2010; Bidarian and Jafari, 2012) and experience job satisfaction (Zynalpoor and Kamaly, 2010). This situation, which is reflected in the results of the research, can be said to be related to the characteristics of the school principals, who are described as agile by the teachers, to meet the expectations of the teachers, and thus to achieve job satisfaction mentally and emotionally. In addition, it is thought that agile school principals’ communication, collaborative approaches, creating a trustworthy environment develop the perception that they are managed fairly in their organization. It can be said that teachers’ high organizational justice perceptions are related to their school life and provide job satisfaction.

Perceived justice and injustice within the organization cause positive and negative emotional reactions in employees (Cohen-Charash and Mueller, 2007; Murphy and Tyler, 2008). More specifically, job satisfaction increases more when the positive relationships between school principals’ agile leadership behaviors and teachers’ job satisfaction are included in the organizational justice factor. In the study, it was remarkable to determine the “full mediation” role of organizational justice in the relationship between the agile leadership characteristics of school principals and teachers’ intrinsic job satisfaction. In other words, the agile leadership characteristics of school principals positively affect teachers’ internal job satisfaction both directly and indirectly through organizational justice. A number of effective leadership skills such as making fair decisions in organizations, participating in the decision-making process (Muhammad, 2004; Eberlin and Tatum, 2008) positively affect the perception of justice of the employees. The leader’s supportive behaviors create a fair sense of organization and more trust on employees (DeConinck, 2010). Thanks to the collaborative, impartial, consistent and fair approach of agile leaders, employees feel strong in uncertain and challenging conditions and strengthen their belief that they will find effective solutions in the face of problems (McKenzie and Aitken, 2012). The fact that leaders have the ability to control both their own emotions and the emotions of others during stress enables employees to develop pleasing behaviors (Mayer and Salovey, 1995; Miao et al., 2016) and to experience high job satisfaction (Ouyang et al., 2015). On the other hand, it has been determined that negative leadership characteristics, which cannot analyze problems in organizations, delay the decision-making process, have difficulty in communicating and do not trust their leadership competence, cause a decrease in organizational justice perceptions of employees (Holtz and Hu, 2017). The flexible and adaptable behaviors, empathy skills, and fast and effective behaviors of agile school principals in the school enable teachers to be more effective in their education-teaching and administrative processes and prevent them from experiencing disappointment. In addition, agile school principals’ encouragement of transparent, collaborative approaches and open and comprehensive communication can improve employees’ understanding of decisions, and the perception that they are treated fairly in all kinds of decisions and work distribution. Because relationship-oriented leadership behaviors affect the perception of organization-oriented justice (Karam et al., 2019), and the perception of justice for the leader and the perception of organizational justice together cause more positive outcomes on teachers. This situation contributes to teachers’ feeling well in the school environment and improving their job satisfaction.

In the research, it has been revealed that organizational justice mediates “partially” in the relationship between the agile leadership characteristics of school principals and external job satisfaction of teachers. To put it differently, the agile leadership characteristics of school principals affect teachers’ external job satisfaction both directly and indirectly through organizational justice. An agile leader with high learning agility has the knowledge and skills to meet the ever-changing business needs and encourages further development by including employees in the learning environment (Cashman, 2011; De Meuse et al., 2012; McKenzie and Aitken, 2012; Drinka, 2018). The continuous learning environment created within the organization affects both leaders and employees to develop new skills (Michinson and Morris, 2014). Effective agile leaders, who learn from their experiences, perform better in management and their collaborative approaches share their employees in the success of the organization (Narel, 2017; Kostrad, 2019). Agile practices and teams developed by agile leaders can cause their employees to provide job satisfaction that will meet their needs (Melnik and Maurer, 2006; Tripp et al., 2016). The ability of high-performing agile leaders to respond quickly to factors such as rewards, promotions, participation in decisions, and wages that will provide external satisfaction (Setili, 2015; Fachrunnisa et al., 2020) can reinforce the belief that material and moral justice will be provided. Ambrose and Schminke (2009) determined that the general perception of justice mediates the relationship between employees’ distributive, procedural and interactional justice judgments and their job satisfaction. The high performance of agile school administrators in their administrative processes, the knowledge of teachers about this process, and meeting their wishes and needs can improve the perception that they are treated equally and fairly. To this extent, teachers can provide more external satisfaction by avoiding unproductive negative behaviors such as leaving work, arriving late, and absenteeism that will put the school in a difficult situation. In addition, according to the research findings, the partial mediating role of organizational justice makes us think that there are other variables in the relationship between agile leadership and external job satisfaction. Finally, the relationships between agile leadership, job satisfaction, and organizational justice behaviors should not be ignored. Accordingly, school principals should keep their direct and indirect effects on organizational justice and job satisfaction in mind in school management practices by developing agile leadership characteristics.

## Conclusion

This research empirically revealed the mediating role of organizational justice in the relationship between school principals’ agile leadership characteristics and teachers’ job satisfaction. As in every research, there are some limitations in our research as well. The first of these is to evaluate the data of the research by obtaining it from a single source (teachers). Future research can use more than one data source and compare the research findings by revealing the concordance between school principals’ agile leadership self-assessments and teachers’ assessments. Secondly, organizational structure may be effective in the relationship between agile leadership and job satisfaction because organizational structure shapes the relationship between leadership style and job satisfaction (McCartney, 1978; Neubert et al., 2016) and affects employee motivation (Worthy, 1950). Because the current research was conducted in schools, findings from different organizational structures may not support our results. This relationship may become more evident in environments where uncertainty and complexity are more struggled, and in organizations that may constantly compete. The third limitation of the study is geographical factors which are an important feature that affects the leadership capacities of school principals (Luo, 2004). The fact that the data were obtained only from teachers working in Istanbul limits the research. With the data to be obtained for school principals in other geographies, school principals in Turkey can be compared and information that will guide education policies can be reached.

This research was conducted during the COVID-19 pandemic. For this reason, it may be necessary to compare the results with the results of the post-pandemic research. Because, during the COVID-19 pandemic period, school principals may not have found a suitable school environment to influence teachers’ perceptions of organizational justice and job satisfaction with agile leadership behaviors, since schools suspended education for a long time.

## Data Availability Statement

The raw data supporting the conclusions of this article will be made available by the authors, without undue reservation.

## Ethics Statement

This study was reviewed and approved by the Ethics Committee of Istanbul Sabahattin Zaim University (Date and Number: 27.05.2021-E7179). Written informed consent was obtained from all participants for their participation in this study. Written informed consent was obtained from the individual(s) for the publication of any potentially identifiable images or data included in this article.

## Author Contributions

MÖ devised the research idea, developed the research model, wrote the method part, ran the analytic calculations, limitations, and implications, checked for the literature and discussion part, and arranged the last version of the manuscript. ŞY wrote the introduction part, performed the results, discussion, recommendations, limitations, and controlled the other parts in terms of language and contextual check for the manuscript. AA data collecting process, data analysis, and wrote the introduction part and discussion. All authors contributed to the article and approved the submitted version.

## Conflict of Interest

The authors declare that the research was conducted in the absence of any commercial or financial relationships that could be construed as a potential conflict of interest.

## Publisher’s Note

All claims expressed in this article are solely those of the authors and do not necessarily represent those of their affiliated organizations, or those of the publisher, the editors and the reviewers. Any product that may be evaluated in this article, or claim that may be made by its manufacturer, is not guaranteed or endorsed by the publisher.
